# Differential Expression of miRNAs in Trichloroethene-Mediated Inflammatory/Autoimmune Response and Its Modulation by Sulforaphane: Delineating the Role of miRNA-21 and miRNA-690

**DOI:** 10.3389/fimmu.2022.868539

**Published:** 2022-03-29

**Authors:** Nivedita Banerjee, Hui Wang, Gangduo Wang, Paul J. Boor, M. Firoze Khan

**Affiliations:** Department of Pathology, University of Texas Medical Branch, Galveston, TX, United States

**Keywords:** miRNA, trichloroethene, inflammation, autoimmunity, sulforaphane

## Abstract

Trichloroethene (TCE), an occupational and ubiquitous environmental contaminant, is associated with the induction of autoimmune diseases (ADs). Although oxidative stress plays a major role in TCE-mediated autoimmunity, the underlying molecular mechanisms still need to be delineated. Altered non-coding RNAs, including the expression of microRNAs (miRNAs), can influence target genes, especially related to apoptosis and inflammation, and contribute to ADs. Therefore, the objective of this study was to delineate the contribution of miRNAs in TCE-mediated inflammatory and autoimmune response. To achieve this, we treated female MRL+/+ mice with TCE (10 mmol/kg in corn oil, i.p., every fourth day) with/without antioxidant sulforaphane (SFN; 8 mg/kg in corn oil, i.p., every other day) for 6 weeks. With the use of miRNA microarray, 293 miRNAs were analyzed, which included 35 miRNAs that were relevant to inflammation and ADs. Among those 35 miRNAs, 8 were modulated by TCE and/or TCE+SFN exposure. TCE treatment led to increased expression of 3 miRNAs and also decreased expression of 3 miRNAs. Interestingly, among the 35 differentially expressed miRNAs, antioxidant SFN modulated the expression of 6 miRNAs. Based on the microarray findings, we subsequently focused on two miRNAs (miRNA-21 and miRNA-690), which are known to be involved in inflammation and autoimmune response. The increases in miRNA-21 and miR-690 (observed using miRNA microarray) were further validated by RT-PCR, and the TCE-mediated increases in miR-21 and miR-690 were ameliorated by SFN treatment. Modulating miR-21 and miR-690 by respective inhibitors or mimics suppressed the expression of NF-κB (p65) and IL-12 in RAW 264.7 cells. Our findings suggest a contributory role of miR-21 and miR-690 in TCE-mediated and its metabolite dichloroacetyl chloride (DCAC)-mediated inflammation and autoimmune response and support that antioxidant SFN could be a potential therapeutic candidate for inflammatory responses and ADs.

## Introduction

Autoimmune diseases (ADs) are characterized by systemic or organ-specific inflammation resulting in tissue damage (1). Systemic lupus erythematosus (SLE), scleroderma, rheumatoid arthritis (RA), and autoimmune hepatitis (AIH) are chronic ADs driven by the innate and adaptive immune system and characterized by increased autoantibodies, induction of inflammatory cytokines, and alteration of genetic/epigenetic responses that affect multiple organs (1, 2). The pathogenesis of ADs is associated with either multiple genetic inclinations or environmental factors (3). The role of these two factors may differ among individuals, but these factors modulate the immune system and produce autoantibodies *via* autoreactive T and B cells that lead to AD progression (2, 4). One of the important factors known to be involved in ADs is exposure to environmental contaminants (4–6). Our previous studies have shown that trichloroethene [trichloroethylene (TCE)], an environmental toxicant, and its metabolites induce an autoimmune response in experimental animals (5, 7–10), and oxidative stress plays an important role in TCE-mediated autoimmunity (5, 9–11). Several pathways relevant to oxidative stress or inflammation have been established to understand the TCE-mediated pathogenesis of ADs (5, 7, 10). Despite significant efforts, there is still a lack of sufficient evidence to understand the underlying molecular mechanisms that can eventually lead to the development of effective therapies against ADs (5, 9–12).

Small non-coding RNAs (ncRNAs), such as microRNAs (miRNAs), are single-stranded, small RNAs (18–22 nucleotides long) that regulate gene expression at the post-transcriptional level. By modulating gene expression, many miRNAs have been shown to regulate several biological processes, including cell development, inflammation, cancer, and autoimmunity (12–15). Recent evidence also shows that miRNAs play a crucial regulatory role in immunity and inflammation (15, 16). Innate immunity and inflammatory responses are used as a self-defense mechanism to eliminate foreign pathogens from the body (5, 17). Dysregulation of miRNAs has been observed in the peripheral blood mononuclear cells (PBMCs) of SLE patients (18). Interestingly, it was shown that AD development in MRL/lpr mice was associated with increased levels of inflammatory miRNA 155 (19). Another study showed that both miR-148a and miR-126 are increased in CD4^+^ T cells of SLE patients and MRL/lpr mice and led to the production of autoreactive T cells (2, 20). Interestingly, two miRNAs, namely, miR-155 and miR-146a, are also relevant to inflammation and regulate cytokines in a different pattern (20). Significant induction of miR-21 expression has been observed in patients occupationally exposed to TCE (21, 22). In addition, miR-21 and miR-690 are also known to regulate several pathways related to inflammation, immune system, cell stress, and several critical genes, including NF-kB (p65) (2, 12, 20, 23). Despite significant information on the involvement of various miRNAs in SLE, essentially nothing is known on their status and role in TCE-mediated autoimmunity. Even though we have clearly established an important role of oxidative stress in TCE-mediated autoimmunity, the interrelationship among oxidative stress, miRNA, and autoimmunity is not known. Similarly, studies have shown the involvement of epigenetics, especially aberrant DNA methylation in CD4^+^ T cells from MRL+/+ mice treated with TCE (24, 25), but the status and contribution of miRNAs in TCE-mediated autoimmunity need to be established. Therefore, in this study, we have attempted to study the response and contribution of the miRNAs, especially miR-21 and miR-690, in TCE-treated female MRL+/+ mice using an experimental condition known to elicit an autoimmune response (7–9) and also made an effort to establish that antioxidant (sulforaphane (SFN)) provides protection by regulating miRNAs.

## Material and Methods

### Cell Culture

Immortalized mouse RAW 264.7 cell line was purchased from the American Type Tissue Collection (ATCC; Manassas, VA, USA). The cells were cultured in complete Dulbecco’s modified Eagle medium (DMEM) media, with 10% heat-inactivated fetal bovine serum (FBS) and 1% penicillin and streptomycin according to the supplier’s guidelines. RAW 264.7 cells were incubated at 37°C and treated with either dichloroacetyl chloride (DCAC; a TCE metabolite, 5 mM) (Sigma-Aldrich, St. Louis, MO, USA) alone or treated first with SFN (10 µM) (Sigma-Aldrich) 1 h prior to incubation with DCAC for 24 h (7).

### Animal Studies

The animal protocol was approved by the Institutional Animal Care and Use Committee (IACUC) of the University of Texas Medical Branch (UTMB), and all the experiments were conducted as per the National Institutes of Health (NIH) guidelines. Female MRL+/+ (Murphy Roths Large; MRL/MpJ) mice (5 weeks old) were obtained from the Jackson Laboratory (Bar Harbor, ME, USA) and kept at the UTMB animal facility under controlled conditions of temperature and humidity with a 12-h light/dark cycle. Mice had free access to food and water *ad libitum* and were acclimatized for a week before starting the treatments. Mice were divided randomly into 4 groups of 6 mice each and designated as control, TCE, SFN, or TCE+SFN treatment groups [TCE (Sigma-Aldrich), 10 mmol/kg in corn oil, i.p., every 4th day; SFN 8 mg/kg in corn oil, i.p., every other day] (7–10, 26, 27). The control animals received an equal volume of corn oil only. The selection of the dose and duration of TCE or SFN exposure was based on previous studies (8–10, 26, 27). MRL+/+ mice were chosen for the study, as these mice are autoimmune-prone where most autoantibodies appear several months after their birth and SLE disease appears late in the second year of their lives (8, 9). This slow development of the disease allows us to evaluate the autoimmune potential of specific agents in relatively young animals. After 6 weeks of treatment, the mice were euthanized under ketamine/xylazine anesthesia, and blood was withdrawn. Major organs were then removed and weighed. Liver portions were snap-frozen in liquid nitrogen and stored at −80°C for future use.

### MicroRNA Microarray Analysis

Microarray assay was performed by a service provider (LC Sciences, Houston, TX, USA). Briefly, total RNA was isolated from the liver samples using the mirVana miRNA Isolation Kit (Applied Biosystems, Foster City, CA, USA). A strand-labeling reaction was carried out with total RNA (1 µg), control oligonucleotides, App-Cap3 (5′-adenylated and 3′-dideoxy oligo-adaptor, Integrated DNA Technologies, Coralville, IA, USA), and T4 RNA ligase (Rnl2tr-K227Q, New England BioLabs, Ipswich, MA, USA) in T4 RNA ligase buffer with PEG 8000 and RNase inhibitors (Promega, Madison, WI, USA). Following the manufacturer’s protocol, the reaction mixture was incubated at 16°C for 16 h and stopped by adding an equal volume of hybridization buffer (28). Finally, fluorescence images were monitored using a laser scanner (GenePix 4000B, Molecular Device, San Jose, CA, USA) and digitized using Array-Pro image analysis software (Media Cybernetics, Rockville, MD, USA). Data were analyzed by first subtracting the background and then normalizing the signals using a LOWESS filter (locally-weighted regression) (29).

### Real-Time PCR

Total RNA from cells and tissues was extracted using the RiboPure™ RNA Purification Kit (Invitrogen, Carlsbad, CA, USA). The design of primer sequencing was done using the Primer3Plus program. The primers were obtained from Integrated DNA Technologies (Coralville, IA, USA), and mRNA expressions of NF-kB (p65), cytokine IL-12, and miR-21 and miR-690 were determined. cDNA was synthesized using iScript™ cDNA synthesis kit (Bio-Rad Laboratories, Hercules, CA, USA). Glyceraldehyde 3-phosphate dehydrogenase (GAPDH) and U6 were used as the endogenous loading controls for mRNA and miRNA, respectively (13, 14). Primer sequences are provided in **Supplementary Table S1**.

### Western Blotting

For the extraction of proteins from cells or liver tissues, radioimmunoprecipitation assay (RIPA) buffer (Cell Signaling Technology, Danvers, MA, USA) with protease inhibitor (Sigma-Aldrich, St. Louis, MO, USA) was used. Thirty micrograms of protein lysates was used for Western blotting analyses. The immunoblots were probed with primary antibodies against, pNF-κB (p-p65) and β-Actin (Cell Signaling Technology, Danvers, MA, USA) (10, 30). The immunoblot images were quantified by densitometry and normalized using loading control protein β-actin.

### Isolation of Splenocytes

Mouse splenocytes were isolated from the spleens of control or TCE-treated mice following an earlier method (17). The spleens were gently crushed in RPMI-1640 medium through a cell strainer. Red blood cells were removed by using Red Cell Lysis buffer (Sigma). Splenocytes were collected by centrifugation and re-suspended in complete RPMI-1640 with 10% FBS.

### Cytokine Array

Mouse cytokine antibody pair-based array spotted on a membrane kit (Abcam, Cambridge, MA, USA) was used to measure the cytokines in the splenocyte protein samples according to the manufacturer’s instructions. Briefly, proteins were extracted from spleen cells of MRL+/+ mice using RIPA buffer with protease inhibitor. Proteins (300 μg) were incubated at 4°C overnight with previously blocked membranes (provided). The membranes were then washed with the washing buffer followed by incubation with biotin-conjugated anti-cytokines at 4°C overnight. After being washed, the membranes were then incubated with streptavidin–horseradish peroxidase (HRP) at 4°C overnight. After being washed, the cytokines on membranes were detected using chemiluminescence detection solution (31).

### Transfection Assay

RAW 264.7 cells were plated separately into 6-well flat-bottom plates at 3 × 10^6^/well in a total volume of 2 ml and incubated at 37°C. RAW 264.7 cells were transfected with miR-21 and miR-690 inhibitors or mimics (20 nm) and negative controls (NCs) (20 nm) by using Lipofectamine 2000 reagent according to the manufacturer’s guidelines (Thermo Fisher Scientific, Waltham, MA, USA). In brief, RAW 264.7 cells were incubated with NC, miR-21, or miR-690 inhibitors or mimics separately for 24 h in reduced serum OPTIMEM media (Invitrogen, Carlsbad, CA, USA). The expressions of NF-κB (p65) and cytokine IL-12 were analyzed (13, 32, 33).

### Dual-Luciferase Assay

RAW 264.7 cells (30,000 cells/100 μl in BPS medium) were plated in 96-well plates and allowed to adhere for 24 h. For transfection, 100 ng of DNA constructs [NF-κB Transient Pack (BPS Biosciences, San Diego, CA, USA) consisting of NF-κB reporter vector + constitutively expressing Renilla luciferase vector or NC reporter (non-inducible luciferase vector + Renilla luciferase vector)] along with 20 nM of miR-690 mimics or respective 20 nM of miRNA control were co-transfected into RAW 264.7 cells using Lipofectamine 2000 (Thermo Fisher Scientific). Luciferase activity was measured 24 h post-transfection with the Dual-Luciferase Reporter Assay System (BPS Biosciences, San Diego, CA, USA) as per the manufacturer’s protocol. Luciferase activity was measured as relative luciferase units (firefly luciferase/Renilla luciferase) (12, 23).

### Histological Examination

Spleen tissue samples were fixed in 10% buffered formalin, dehydrated, and paraffin embedded. These paraffin sections were stained with H&E (17, 30). Images were taken using an Olympus 1X71 microscope (Olympus, Hamburg, Germany).

### Statistical Analysis

The values are presented as means ± SEM. The data were analyzed using a one-way ANOVA followed by the Tukey–Kramer multiple comparisons test. An unpaired Student’s t-test was performed to find out whether there is a significant difference between the two groups (control vs. TCE). The p-values <0.05 were considered statistically significant.

## Results

### Differential Expression of Micro-RNAs in the Liver of Trichloroethene-Treated MRL+/+ Mice

**Figure 1:**
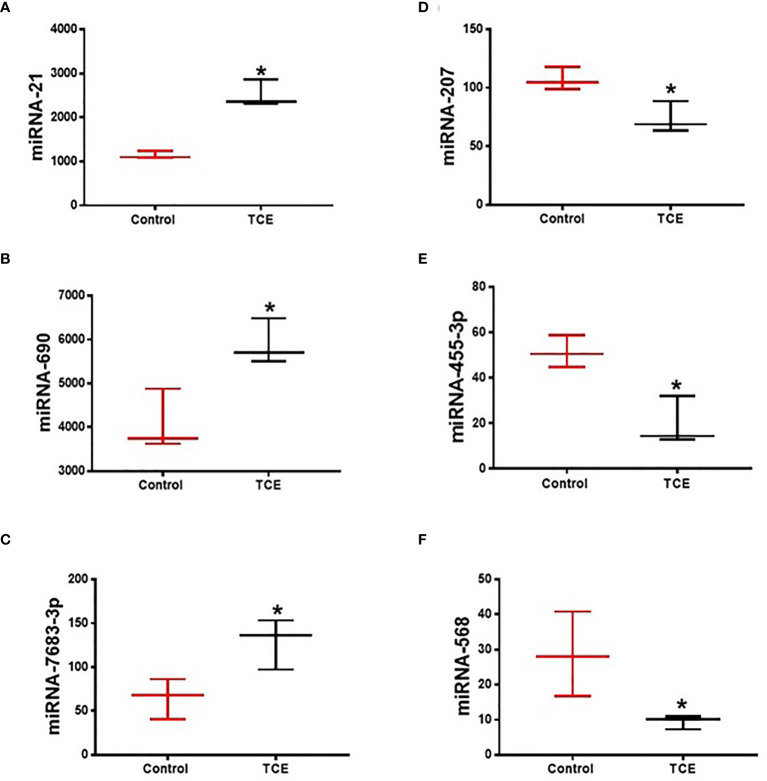
TCE exposure modulated the expression of microRNAs in the livers of MRL+/+ mice as determined by miRNA microarray. **(A–F)** mmu-miR-21a-5p, mmu-miR-690, mmu-miR-7683-3p, mmu-miR-207, mmu-miR-455-3p, and mmu-miR-568. n = 3 in each group. *p ≤ 0.05 vs. controls. TCE, trichloroethene.

**Figure 2:**
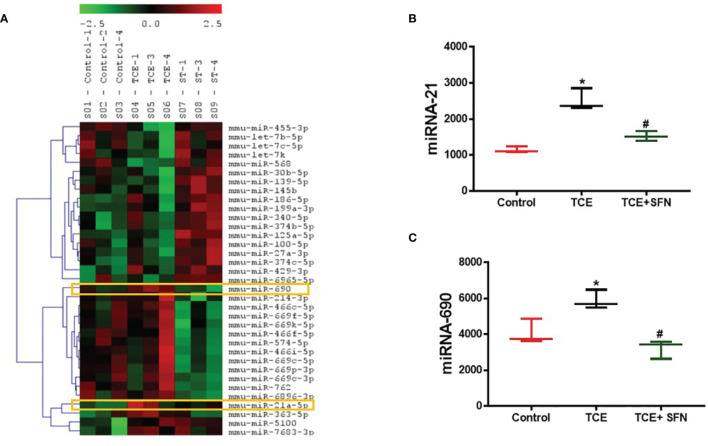
Differential expression of microRNA as determined by miRNA microarray in the livers of MRL+/+ mice. **(A)** Modulation of microRNAs in the livers of MRL+/+ mice following TCE and/or TCE+SFN treatment. **(B, C)** TCE exposure led to increased miR-21 and miR-690 expression, whereas SFN treatment ameliorated the effect of TCE. n = 3 in each group. *p < 0.05 vs. controls; ^#^p < 0.05 vs. TCE-treated mice. TCE, trichloroethene; SFN, sulforaphane.

The regulatory role of miRNAs in the development and normal function of immune cells involved in innate immunity is well established. MiRNAs are also involved in the adaptive immune system *via* activation and development of reactive T cells and B cells responding to specific antigens (20). In this study, we chose autoimmune-prone MRL+/+ mice to identify the differentially expressed miRNAs in the liver following TCE exposure. To explore the effect of TCE on the miRNA profile, we first screened for all the miRNAs in the livers of TCE-treated mice. In the microarray analysis, out of 293 miRNAs that were evaluated, 6 miRNAs were modulated by TCE. Out of 6 miRNAs, 3 were increased (mmu-miR-21a-5p, mmu-miR-690, and mmu-miR-7683-3p), whereas 3 miRNAs were downregulated (mmu-miR-207, mmu-miR-455-3p, and mmu-miR-568) (**Figure 1** and **Table 1**). We further evaluated 35 miRNAs relevant to inflammation and ADs (**Figure 2A**) following TCE and/or TCE+SFN treatment. Out of those 35 miRNAs, 8 miRNAs were modulated (mmu-miR-21a-5p, mmu-miR-690, mmu-miR-125a-5p, mmu-miR-139-5p, mmu-miR-455-3p, mmu-miR-568, mmu-miR-5100, and mmu-miR-145b; **Figure 2A** and **Table 2**). Interestingly, among these differentially expressed miRNAs, antioxidant SFN ameliorated the expression of 6 miRNAs (mmu-miR-21a-5p, mmu-miR-690, mmu-miR-139-5p, mmu-miR-455-3p, mmuwfi 2-miR-568, and mmu-miR-145b; **Figure 2A** and **Table 2**).

**Table 1:** Differential expression of microRNAs as determined by miRNA microarray following TCE treatment.

Reporter name	p-Value	Target sequence (5′ to 3′)
mmu-miR-21a-5p	0.002	UAGCUUAUCAGACUGAUGUUGA
mmu-miR-690	0.022	AAAGGCUAGGCUCACAACCAAA
mmu-miR-7683-3p	0.0421	UGGAAAGGUGGAACACGGAAC
mmu-miR-207	0.0243	GCUUCUCCUGGCUCUCCUCCCL
mmu-miR-455-3p	0.0125	GCAGUCCACGGGCAUAUACAC
mmu-miR-568	0.053	AUGUAUAAAUGUAUACACAC
mmu-miR-363-5p	0.072	CAGGUGGAACACGAUGCAAUUU
mmu-let-7b-5p	0.0767	UGAGGUAGUAGGUUGUGUGGUU
mmu-let-7c-5p	0.0912	UGAGGUAGUAGGUUGUAUGGUU
mmu-let-7a-5p	0.0938	UGAGGUAGUAGGUUGUAUAGUU
mmu-let-7d-5p	0.0981	AGAGGUAGUAGGUUGCAUAGUU

p ≤ 0.05 vs. TCE.

TCE, trichloroethene.

**Table 2:** Differential expression of microRNAs as determined by miRNA microarray following TCE or TCE+SFN treatment.

Reporter name	p-Value	Target sequence (5′ to 3′)
mmu-miR-21a-5p	0.0047	UAGCUUAUCAGACUGAUGUUGA
mmu-miR-690	0.0036	AAAGGCUAGGCUCACAACCAAA
mmu-miR-125a-5p	0.0029	UCCCUGAGACCCUUUAACCUGUGA
mmu-miR-139-5p	0.0065	UCUACAGUGCACGUGUCUCCAG
mmu-miR-455-3p	0.0075	GCAGUCCACGGGCAUAUACAC
mmu-miR-568	0.0171	AUGUAUAAAUGUAUACACAC
mmu-miR-5100	0.0439	UCGAAUCCCAGCGGUGCCUCU
mmu-miR-145b	0.0447	GUCCAGUUUUCCCAGGAGACU

p < 0.05 (comparison among 3 groups: control, TCE, and TCE+SFN).

TCE, trichloroethene; SFN, sulforaphane.

### Identification of Inflammation- and Autoimmune Disease-Related miRNAs

**Figure 3:**
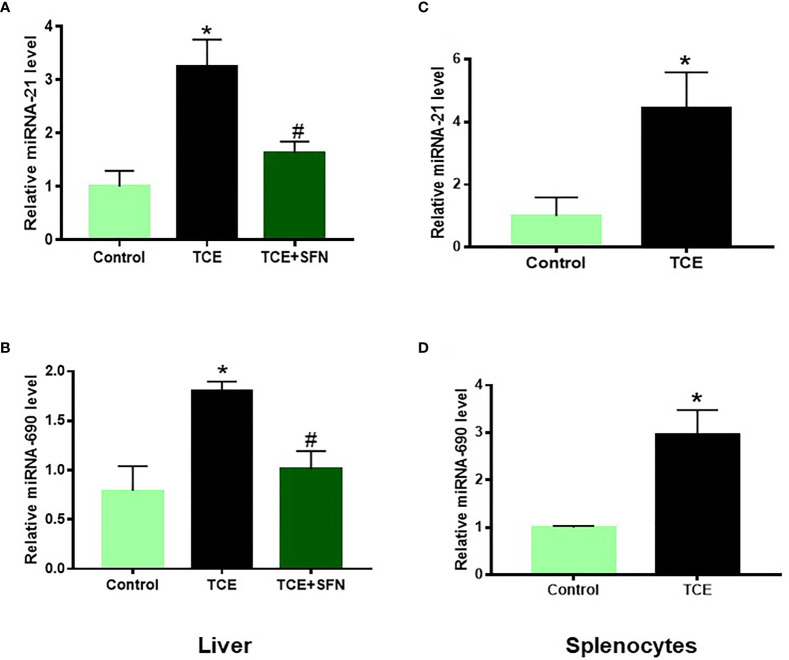
TCE-mediated increased miR-21 and miR-690 in the livers of MRL+/+ mice as determined by RT-PCR. **(A, B)** TCE treatment led to increased expression of miR-21 and miR-690 in the livers of MRL+/+ mice, and SFN treatment ameliorated the effect of TCE. **(C, D)** Increased expression of miR-21 and miR-690 in the splenocytes of TCE-treated MRL+/+ mice. n = 4 in each group. *p < 0.05 vs. controls; ^#^p < 0.05 vs. TCE-treated mice. TCE, trichloroethene.

Out of 35 differentially modulated miRNAs, as evident from microarray analysis, we selected mmu-miR-21a-5p (miR-21) and mmu-miR-690 (miR-690) as the top candidates for further investigation (**Figures 2A–C**), as both these miRNAs not only showed ~2-fold induction in TCE-treated mice compared to the controls but also are due to their potential role in inflammatory responses (12, 34). Further validation of the increased miR-21 and miR-690 levels upon TCE treatment was done by RT-PCR, which also showed significant induction in the expression of miR-21 and miR-690 in the livers (**Figures 3A, B**). Our previous studies with an antioxidant SFN showed significant anti-inflammatory effects as well as amelioration of TCE-mediated autoimmune response (7). To determine if SFN can also provide protection through the modulation of miRNAs, we analyzed these miRNAs with or without SFN treatment along with TCE. As evident from **Figures 2B, C**, **3A, B**, SFN treatment attenuated the effect of TCE by suppressing the expression of both miR-21 and miR-690 in the liver. Interestingly, these miRNAs (miR-21 and miR-690) were also significantly increased in the splenocytes of TCE-treated mice (**Figures 3C, D**).

### Trichloroethene-Mediated Inflammatory Responses in the Spleen

**Figure 4:**
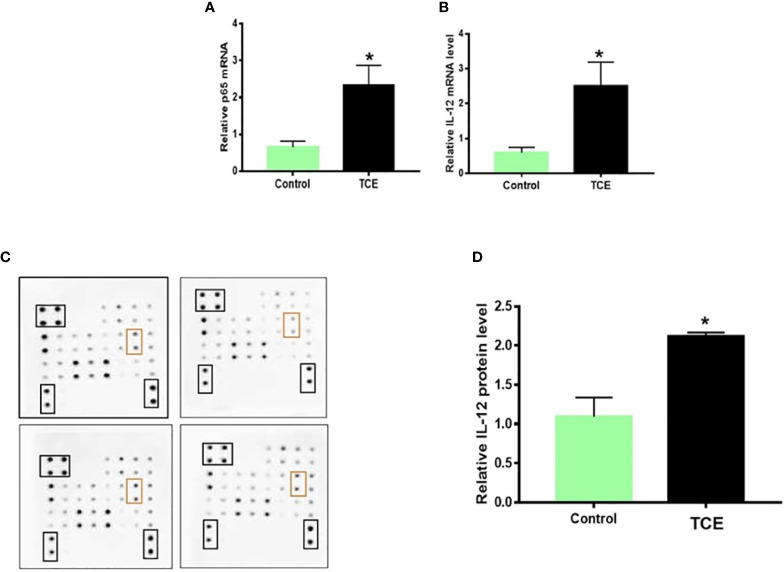
TCE exposure led to increased NF-kB (p65) and IL-12 expression in the splenocytes of MRL+/+ mice. **(A, B)** NF-kB (p65) and IL-12 mRNA expression in the splenocytes. **(C, D)** Protein expression of cytokines and densitometry of IL-12 levels in the splenocytes. n = 4 in each group. *p < 0.05 vs. controls. TCE, trichloroethene.

To establish an association of the changes in miRNA levels with the inflammatory response, we determined the inflammatory markers in the splenocytes of TCE-treated mice. TCE treatment led to significantly increased mRNA expression of NF-kB and IL-12, and protein levels of IL-12 (**Figures 4A–D**).

### Trichloroethene Exposure Leads to Lymphocytic Infiltration in the Spleen

**Figure 5:**
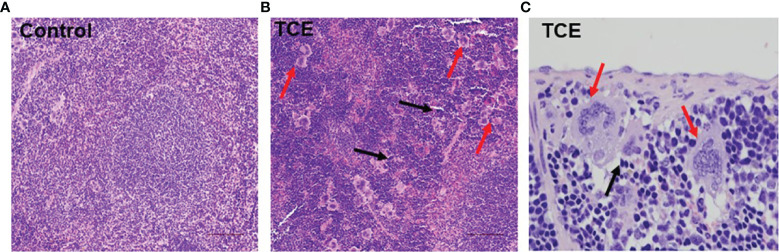
TCE treatment resulted in macrophage-like giant cells and lymphocytic infiltration in the spleens. **(A)** Normal architecture of spleen. **(B)** Red arrows indicate infiltration of macrophage-like giant cells, and black arrows point to lymphocytic infiltration in the spleens of TCE-treated mice (×20 magnification). **(C)** Giant macrophage-like cells (red arrows) in TCE-treated mice (×40 magnification). TCE, trichloroethene.

Infiltration of immune cells in the liver, spleen, and kidney is associated with AIH or SLE phenotype (17, 30, 35). Based on H&E data, an abundant cluster of large macrophage-like cells and lymphocytic infiltration (pointed by red and black arrows, respectively) were seen in the spleens of TCE-treated mice (**Figure 5**).

### Regulatory Effect of MiR-21 on Potential Target Genes

**Figure 6:**
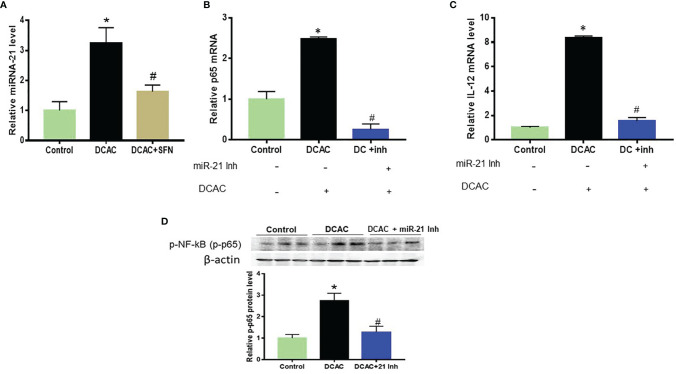
DCAC treatment led to increased miR-21 expression in RAW 264.7 cells. **(A)** Increased expression of miR-21 in the DCAC-treated RAW 264.7 cells and its amelioration by SFN. **(B, C)** mRNA expression of NF-κB (p65) and IL-12 in RAW 264.7 cells transfected with miR-21 inhibitors. n = 4 in each group. **(D)** Protein expression of NF-κB (p65) and its densitometry analysis in RAW 264.7 cells following DCAC treatment and transfection with miR-21 inhibitors. n = 3 in each group. *p < 0.05 vs. controls; ^#^p < 0.05 vs. DCAC-treated RAW 264.7 cells. DCAC, dichloroacetyl chloride; SFN, sulforaphane.

To further verify the findings with TCE exposure *in vivo*, we determined these miRNAs (miR-21 and miR-690) *in vitro* using macrophages (RAW 264.7 cells) (due to infiltration of macrophage-like cells that were found in the spleen; H&E staining, **Figure 5**) after their incubation with DCAC, a highly reactive metabolite of TCE with a potential to generate both inflammatory and autoimmune response (8, 36). DCAC treatment also significantly increased miR-21 expression in RAW 264.7 cells, which was attenuated by SFN (**Figure 6A**). To evaluate the target genes of miR-21 in TCE-mediated inflammation responses, RAW 264.7 cells were transfected with miR-21 mimics or inhibitors (20 nM) or respective NC. Transfection efficacy with miR-21 mimics showed significantly higher expression of miR-21 as compared to the NC group (data were not shown). MiR-21 inhibitors suppressed the effect of DCAC-mediated inflammation by suppressing p-NF-KB and IL-12 expression (**Figures 6B–D**). It is thus evident that miR-21 inhibitors can modulate mRNA expression for NF-kB (p65) and IL-12 in RAW 264.7 cells. Our data also suggest that miR-21 could play an important role in DCAC-mediated inflammation and could be a potential therapeutic target for TCE-mediated ADs.

### Regulatory Effect of MiR-690 on Potential Target Gene

**Figure 7:**
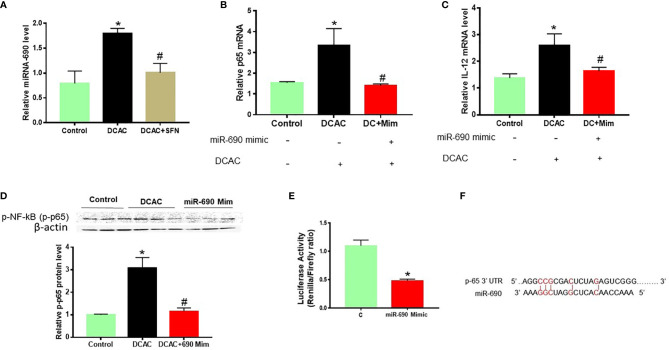
DCAC treatment led to increased expression of miR-690 in RAW 264.7 cells. **(A)** Increased expression of miR-690 in the DCAC-treated RAW 264.7 cells. SFN treatment ameliorated the effect of DCAC. **(B, C)** mRNA expression of NF-κB (p65) and IL-12 in RAW 264.7 cells transfected with miR-690 mimics. n = 4 in each group. **(D)** Protein expression of NF-κB (p65) and its densitometry analysis in RAW 264.7 cells. **(E)** NF-κB-luciferase reporter plasmid along with miR-690 mimics or control were co-transfected into RAW 264.7 cells. **(F)** Predicted miR-690 targeting region in 3′ UTR of NFkB. Luciferase activity is the ratio of Renilla/*Firefly*. Renilla is raw renilla luciferase activity, whereas Firefly is firefly luciferase activity. The 3′ UTR starts at bp 6,577 and ends at bp 6,627. n = 3 in each group. *p < 0.05 vs. controls; ^#^p < 0.05 vs. DCAC-treated RAW 264.7 cells. DCAC, dichloroacetyl chloride; SFN, sulforaphane.

In our previous study, we have shown that upregulation of p38 MAPK/NF-kB (p65) plays an important role in TCE-mediated autoimmunity (30). Cytokines play a crucial role in the development of inflammatory responses and ADs (37). To evaluate the target gene of miR-690 in TCE-mediated inflammation and autoimmunity, mouse RAW 264.7 cells were treated with miR-690 mimics or respective NC. Like miR-21, SFN also ameliorated DCAC-induced miR-690 expression (**Figure 7A**). MiRNA-690 mimics suppressed the expression of p-NF-kB and IL-12 in RAW 264.7 cells in the presence of DCAC (**Figures 7B–D**). Taken together, DCAC treatment induced miR-690 in RAW 264.7 cells, and these findings suggest that manipulation of miR-690 could be useful in modulating DCAC-induced inflammatory markers.

To obtain further evidence that NF-κB (p65) is a direct target of miR-690, a dual-luciferase reporter assay was used to determine the influence of miR-690 on signaling NF-κB transcriptional activation. NF-κB-luciferase reporter plasmid along with miR-690 mimics or control was co-transfected into RAW 264.7 cells. We observed that miR-690 mimics markedly inhibited the luciferase activity of cells (**Figures 7E, F**).

## Discussion

Previous studies have shown several intrinsic (age, sex, and genetics) and extrinsic (environmental chemicals, toxicants, etc.) factors can play critical roles in the pathogenesis of ADs (4, 6). TCE, a ubiquitous environmental contaminant, has been involved in the development of autoimmune disorders in humans (38–41) and in experimental animals (4, 7, 8, 30, 42–45). Previous studies have shown aberrant DNA methylation in CD4^+^ T cells isolated from TCE-mediated MRL+/+ mice (25, 46). However, no effort has been made to explore the contribution of miRNAs in TCE-induced autoimmunity. Therefore, this study was focused on determining the involvement and contribution of miRNAs in TCE-treated MRL+/+ mice using an experimental condition known to elicit an autoimmune response (8, 10, 30). MiRNAs are important biomarkers in the diagnosis of cancer and play a major role in the development of hematopoietic lineage (47). Recent studies indicated that miRNA can also be used as biomarkers for ADs. Increasing evidence suggests that the regulatory roles of miRNAs are complicated, as one miRNA can target different mRNAs, which control different physiological responses. In this study, out of 35 miRNAs related to inflammation and autoimmunity, 8 miRNAs showed aberrant regulation, including 3 miRNAs that were increased, whereas 3 were decreased following TCE treatment. Among the increased miRNAs, we focused on miR-21 and miR-690 for further evaluation based on their potential role in inflammation and ADs (2, 12, 23). Our microarray data also showed antioxidant SFN ameliorated expression of 6 miRNAs (mmu-miR-21a-5p, mmu-miR-690, mmu-miR-139-5p, mmu-miR-455-3p, mmu-miR-568, and mmu-miR-145b).

Emerging studies indicate that miR-21 promotes inflammation and plays an important role in the pathogenesis of ADs. The silencing of miR-21 significantly attenuated the severity of ADs in an experimental model (34, 48). It is obvious from studies that miR-21 is one of the most predominantly dysregulated miRNAs in different inflammatory diseases (47, 49, 50). Increased expression of miR-21 in T lymphocytes is associated with ADs such as SLE, RA, and multiple sclerosis (MS) (47, 50). MiR-21 expression is particularly increased in activated T cells rather than non-activated T cells or antigen-presenting cells (50). Studies have shown that phorbol 12-myristate 13-acetate (PMA), cytokines like IL-4 or IL-6, and lipopolysaccharide (LPS) can induce the expression of miR-21 in mammalian monocytes (49, 50), suggesting that miR-21 can be used as an important biomarker for inflammatory response. It was also observed that LPS, PMA, and IL-6 induced miR-21 expression through the activation of such transcription factors as NF-kB and AP-1 (50). MiR-21 controls various cell functions such as T-cell activation and apoptosis, Th1/Th2 balance, and DNA hypomethylation (50). MiR-21 was also shown to be significantly increased in the plasma, peripheral blood CD4+ mononuclear cells, B cells, and T cells in the patients with SLE or splenic CD4^+^ T cells of MRL/lpr mice (2). Transcription factor NF-κB is identified as a redox sensor and also functions as a master regulator of the inflammatory response to influence both innate and adaptive immune functions (51). The fact that increased expression of miRNA-21 induced the production of superoxide anions and ROS generation (52) suggests a close association among miRNA-21, NFκB, and oxidative stress. Similarly, miR-690 controls inflammation and oxidative stress at the post-transcriptional level. Interestingly, TCE-treated MRL+/+ mice in this study showed significantly increased expression of both miR-21 and miR-690 in the liver and splenocytes, as well as in DCAC-treated RAW 264.7 cells. Importantly, SFN treatment suppressed the expression of both miR-21 and miR-690 in mice treated with TCE as well as in RAW 264.7 cells treated with DCAC. Earlier, we have reported that SFN ameliorates TCE-mediated inflammatory and autoimmune responses *via* downregulation of NF-kB and upregulation of Nrf2 (7, 30). Here, we further report that SFN-mediated amelioration of TCE effect in MRL+/+ mice is associated with reduced levels of miR-21 and miR-690. This also suggests that an antioxidant-like SFN can exert anti-inflammatory functions by regulating miRNAs like miRNA-21 and miRNA-690.

The regulatory role of miRNAs for targeting certain cell signaling pathways can be cell or tissue specific. NF-κB (p65) plays an important role in pro-inflammatory signaling and leads to cytokine/chemokine production, which can influence the progression of TCE-mediated ADs (7, 30). MiR-21 has been shown to have a rather complicated role in the activation of NF-κB (p65) signaling in various cell types (47, 53). In our previous study, we showed that DCAC treatment increased the expression of NF-κB (p-65) in LPS-activated Kupffer cells and TCE-treated MRL+/+ mice (7). In this study, DCAC-treated RAW 264.7 cells and splenocytes isolated from TCE-treated mice showed increased miR-21 expression as well as NF-κB (p-65), indicating a potential role of miR-21 in TCE-mediated ADs. However, miR-21 could play a dual role in influencing the NF-κB pathway—it might inhibit or activate NF-κB signaling (53, 54), depending on cell types and diseases. Interestingly, NF-κB (p65) activation can also influence the transcription of miR-21 and the production of several pro-inflammatory cytokines (55). MiR-21 has been identified as one of the important markers for immune cell activation and functions as a direct target of NF-κB (50, 55–57). On the other hand, LPS or IL-6 can induce miR-21 through activation of transcription factors such as NF-κB (50). As a result, miR-21 can either inhibit or activate the expression of the NF-kB (p65) signaling pathway in different target genes and disease models. In this study, miR-21 inhibitors decreased the effect of DCAC-mediated inflammation by suppressing p-NF-KB or IL-12 expression in RAW 264.7 cells. Thus, results from our study show that miR-21 is linked to NF-kB activation in the presence of TCE/DCAC.

Like miR-21, miR-690 is also known to regulate several pathways related to inflammation, cell stress, and several critical genes such as NF-kB (p65) and MAPKs (12, 23). In this study, TCE/DCAC significantly increased miR-690 in mouse liver and splenocytes and RAW 264.7 cells. Interestingly, miR-690 mimics suppressed the expression of p-NF-kB (p65) and IL-12 in DCAC-treated RAW 264.7 cells. MiR-690 mimics markedly inhibited the luciferase activity of RAW 264.7 cells. Thus, our data suggest that NF-kB is one of the important targets of both miR-21 and miR-690, which can further influence the downstream pro-inflammatory cytokines in TCE-mediated ADs. Overall results suggest that miR-21 inhibitors block DCAC-mediated inflammation, and miR-690 mimics target p65 through inhibition. To further understand the complicated role of miR-21 and miR-690 in TCE-mediated autoimmunity, more studies are warranted, especially using miR-21 or miR-690 knock-out mice.

In conclusion, our data show that TCE/DCAC treatment increases miR-21 and miR-690 expression in mouse liver, splenocytes, and RAW 264.7 cells. SFN treatment ameliorated the TCE-induced differential expression of several miRNAs, including miR-21 and miR-690. Our data suggest NF-kB (p65) as one of the pro-inflammatory target genes of miR-21 and miR-690. Furthermore, miR-21 and miR-690 *via* the use of respective inhibitors/mimics modulated the expression of NF-kB (p65) and pro-inflammatory cytokines. Our findings suggest a contributory role of miRNAs, especially the contribution of miR-21 and miR-690 in TCE-mediated autoimmunity. Furthermore, protection provided by SFN on differential expression of miRNAs could lead to novel opportunities for the prevention and treatment of ADs. Future studies on the status and in-depth analysis of miRNA-mediated functional changes, especially during the initiation and progression of the TCE-mediated inflammation and autoimmunity, are necessary to establish a definite role of these miRNAs.

## Data Availability Statement

The original contributions presented in the study are publicly available. This data can be found here: NCBI repository, GSE197615.

## Ethics Statement

The animal study was reviewed and approved by IACUC, UTMB Galveston.

## Author Contributions

NB designed and conducted the study and participated in manuscript writing. MFK designed the study and participated in manuscript writing. HW, GW, and PB conducted part of the study and helped in editing the manuscript. All authors listed have made a substantial, direct, and intellectual contribution to the work and approved it for publication.

## Funding

This work was supported by R01 Grants ES026887 and ES016302 from the National Institute of Environmental Health Sciences (NIEHS), NIH.

## Author Disclaimer

Its contents are solely the responsibility of the authors and do not necessarily represent the official views of the NIEHS, NIH.

## Conflict of Interest

The authors declare that the research was conducted in the absence of any commercial or financial relationships that could be construed as a potential conflict of interest.

## Publisher’s Note

All claims expressed in this article are solely those of the authors and do not necessarily represent those of their affiliated organizations, or those of the publisher, the editors and the reviewers. Any product that may be evaluated in this article, or claim that may be made by its manufacturer, is not guaranteed or endorsed by the publisher.
