# Supplementary material for: Differential Expression of miRNAs in Trichloroethene-Mediated Inflammatory/Autoimmune Response and Its Modulation by Sulforaphane: Delineating the Role of miRNA-21 and miRNA-690
Source: Front Immunol. 2022 Mar 29;13:868539. doi: 10.3389/fimmu.2022.868539 (PMC9001960; doi:10.3389/fimmu.2022.868539)
Supplement: Supplementary file 1 [file Table_1.docx]

**Table S1**

**Real-Time PCR primer sequences for mouse gene**

**Gene names Sequences**

**NFkB (p65)** F: TTT TCG ACT ACG CAG TGA CG

R**:** CCA AGT GCA GAG GTG TCT GA

**IL-12**  F: AAG GAA CAG TGG GTG TCC AG

R: CAT CTT CTT CAG GCG TGT CA

**Target Sequence of micro-RNAs**

**Gene names Target Sequence (5’-3’)**

| mmu-miR-690 |  | AAAGGCUAGGCUCACAACCAAA |
| --- | --- | --- |

mmu-miR-21a-5p UAGCUUAUCAGACUGAUGUUGA

Mimic and inhibitor sequences are provided by Thermo Fisher Scientific

**mmu-miR-690 mimic MC11517**

Sense complement (5'-3'): UGGUUGUGAGCCUAGCCUUUTT

antisense miRNA (5'-3'): AAAGGCUAGGCUCACAACCAAA

**mmu-miR-690 inhibitor MH11517**

Sense complement (5'-3'): UUUGGUUGUGAGCCUAGCCUUU

**mmu-miR-21a-5p mimic MC10206**

Sense complement (5'-3'): AACAUCAGUCUGAUAAGCUATT

antisense miRNA (5'-3'): UAGCUUAUCAGACUGAUGUUGA

**mmu-miR-690 inhibitor MH10206**

Sense complement (5'-3'): UCAACAUCAGUCUGAUAAGCUA
